# Role of Microenvironmental Components in Head and Neck Squamous Cell Carcinoma

**DOI:** 10.3390/jpm13111616

**Published:** 2023-11-17

**Authors:** Enar Jumaniyazova, Anastasiya Lokhonina, Dzhuliia Dzhalilova, Anna Kosyreva, Timur Fatkhudinov

**Affiliations:** 1 Research Institute of Molecular and Cellular Medicine, Peoples’ Friendship University of Russia (RUDN University), 6 Miklukho-Maklaya Street, 117198 Moscow, Russia; lokhonina-av@rudn.ru (A.L.); kosyreva.a@list.ru (A.K.); fatkhudinov-tkh@rudn.ru (T.F.); 2 Avtsyn Research Institute of Human Morphology of FSBSI Petrovsky National Research Centre of Surgery, 3 Tsyurupy Street, 117418 Moscow, Russia; 3 National Medical Research Center for Obstetrics, Gynecology and Perinatology Named after Academician V.I. Kulakov of Ministry of Healthcare of Russian Federation, 4 Oparina Street, 117997 Moscow, Russia

**Keywords:** head and neck squamous cell carcinoma, HNSCC, tumor microenvironments, hypoxia in the TME, cancer-associated fibroblasts, vascular component, extracellular matrix

## Abstract

Head and neck squamous cell cancer (HNSCC) is one of the ten most common malignant neoplasms, characterized by an aggressive course, high recurrence rate, poor response to treatment, and low survival rate. This creates the need for a deeper understanding of the mechanisms of the pathogenesis of this cancer. The tumor microenvironment (TME) of HNSCC consists of stromal and immune cells, blood and lymphatic vessels, and extracellular matrix. It is known that HNSCC is characterized by complex relationships between cancer cells and TME components. TME components and their dynamic interactions with cancer cells enhance tumor adaptation to the environment, which provides the highly aggressive potential of HNSCC and resistance to antitumor therapy. Basic research aimed at studying the role of TME components in HNSCC carcinogenesis may serve as a key to the discovery of both new biomarkers–predictors of prognosis and targets for new antitumor drugs. This review article focuses on the role and interaction with cancer of TME components such as newly formed vessels, cancer-associated fibroblasts, and extracellular matrix.

## 1. Introduction

Head and neck squamous cell carcinoma (HNSCC) is the seventh most common cancer worldwide, causing more than 660,000 new cases and 325,000 deaths annually [1,2]. Modifiable risk factors for this pathology include tobacco use in one form or another, alcoholic beverages, human papillomavirus (HPV) infection (more commonly associated with oropharyngeal cancer), and Epstein–Barr virus (EBV) infection (especially for nasopharyngeal cancer). People in some countries eat areca nut, which also increases the risk of HNSCC. Previously, HNSCC was classified according to tumor location (tumor of the oral or nasal cavity, oropharynx, nasopharynx, larynx, or hypopharynx), TNM (tumor, nodus, and metastasis) stage, and histology, but the recognition of molecular genetic profiles now allows it to be more accurately classified into individual subtypes [3,4]. Today, the updated TNM classification (eighth edition, 2018), which has several differences from the previous seventh edition of 2010, should be used for staging this nosology. The main changes in HNSCC staging include adding depth of invasion for oral cavity tumors, introducing a pathomorphologic and clinical staging system for high-risk oropharyngeal tumors associated with papillomavirus infection (HPV+), and considering tumor extension beyond the lymph node capsule in high-risk HPV-negative oropharyngeal tumors and head and neck squamous cell carcinoma in other localizations, excluding nasopharyngeal cancer. Assessing the depth of invasion of oral cancer has prognostic value: deeper tumors show an increased risk of metastasis to lymph nodes and a decreased overall survival rate [5]. Extranodal extension, in turn, also serves as an unfavorable prognostic factor for HNSCC, with the exception of HPV+-related tumors [6]. A separate chapter in the eighth edition is devoted to nasopharyngeal cancer. The main changes are the inclusion of a T0 category for patients with metastatic cervical lymph nodes, EBV-positive patients with an unknown primary focus, and changes in the definition of regional lymph nodes. Unlike other localizations of head and neck cancer, for which surgery plays an important role in primary treatment, squamous cell cancer of the nasopharynx is primarily treated with radiation therapy, with or without chemotherapy. For this reason, pathologic classification is irrelevant in this disease of the nasopharyngeal region [7]. In 2022, the fifth edition of the World Health Organization (WHO) classification of head and neck tumors was published, focusing on the distinctive molecular genetic characteristics of head and neck tumors [8]. The delineation of the distinct molecular genetic signatures of HNSCC allows for a significant increase in diagnostic accuracy and also has prognostic value, which, in turn, leads to a personalized treatment approach for each patient. A recent review [9] details the results of the updated WHO classification for squamous HNSCC.

There are many approaches to the treatment of HNSCC, but none of them are effective enough. In the early stages of the tumor (Stages I and II), surgical removal and radiation therapy are highly effective, but 70% of patients are diagnosed with later stages of the disease—III or IV, where the effectiveness of these methods of therapy sharply decreases [10,11]. The ineffectiveness of therapy in a number of patients dictates the need for a deeper understanding of the mechanisms of the pathogenesis of head and neck tumors. To solve this problem, many studies have been conducted to study the properties and relationships between tumor cells and the microenvironment. HNSCC is characterized by complex relationships between stromal, epithelial, and immune cells in the tumor microenvironment (TME) [12,13,14]. The HNSCC TME includes various cells, both stromal and immune, as well as the extracellular matrix (ECM), blood, and lymphatic vessels [13,15]. Recently, a number of authors have emphasized the role of stromal cells in the induction and maintenance of tumors [16,17,18] and have also shown the relationship stromal cells have with the resistance of tumor cells to therapy [19]. We previously described, in detail, the role of immune cells in the development of head and neck tumors [20]. The current review discusses components of the tumor microenvironment such as neovasculature, hypoxia, cancer-associated fibroblasts (CAFs), and the ECM. By combining the accumulated knowledge of researchers around the world in this field, we hope to provide a better understanding of the interaction between HNSCC and its microenvironment to contribute to the discovery of novel biomarkers and targets for antitumor agents.

## 2. Vascular Component of the HNSCC TME

**Figure 1:**
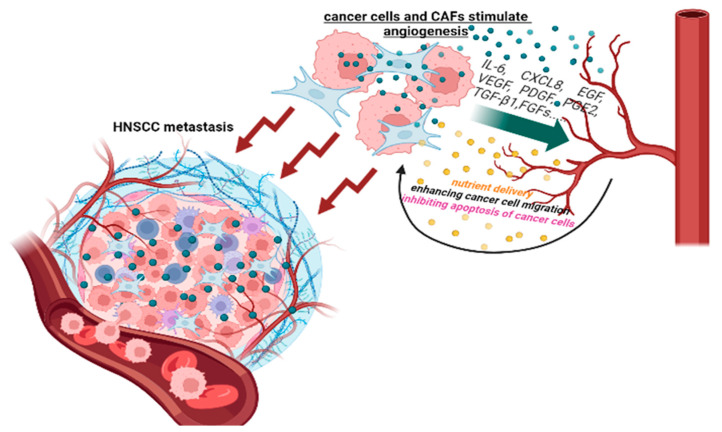
Metastasis of HNSCC as a result of the dynamic interaction of the tumor with blood vessels.

The formation of new vessels, or angiogenesis, is one of the signs of the tumor process [21] and is critical both for the growth of the primary tumor and for the development of distant metastases [22]. Tumor angiogenesis and neovascularization are structurally and functionally different from healthy angiogenesis, with tumor vessels having blunt ends and poor perfusion [23]. Tumor endothelial cells have numerous ruptures, which contribute not only to blood leakage but also to the formation of blood clots and tissue swelling [24]. Various factors are involved in the formation of blood vessels in the TME, such as vascular endothelial growth factor (VEGF), platelet-derived growth factor (PDGF), interleukin-8 (IL-8), delta-like ligand 4 (Dll4), and transforming growth factor families (such as TGF-β) [25,26] (Figure 1).

*VEGF* is a hypoxia-dependent gene and a key factor in tumor vascularization [27] that plays a crucial role in the regulation of blood vessel formation and maintenance. It belongs to the *PDGF* superfamily, which also includes VEGF-A, VEGF-B, VEGF-C, VEGF-D, VEGF-E, and the placental growth factor (PlGF) [28,29]. Each member of the family has its own sphere of action. The most well studied is VEGF-A, which induces angiogenesis and is involved in various physiological and pathological processes, including the growth of malignant neoplasms. VEGF-A initiates the proliferation, migration, and tube formation of endothelium. In already formed vessels, it increases the permeability of the wall, allowing proteins, growth factors, and immune cells to penetrate tissues [30]. The functional potential of VEGF-B in cancer is less pronounced: it can enhance blood vessel growth, improve tissue perfusion, and protect against tissue damage under conditions of severe hypoxia [31]. VEGF-B can also interact with co-receptors called neuropilins (NRP-1 and NRP-2). VEGF-C and VEGF-D are associated with the process of lymphangiogenesis, the formation of lymphatic vessels. Thus, the overexpression of these ligands will promote the metastasis of cancer cells to lymph nodes. The last member of the VEGF family is PlGF, which becomes active in tumors under hypoxic conditions. It also promotes the infiltration and activation of macrophages into tumor tissue, which in turn release pro-inflammatory and pro-angiogenic cytokines such as IL-1 and TNF-α [32]. The VEGF family of ligands plays its role through the cell surface receptor tyrosine kinases, VGFR-1, VGFR-2, and VGFR-3 [33,34]. In addition, as mentioned above, VEGF interacts with NRP-1 and NRP-2 [35,36,37], which both enhance the association between VEGF and its receptors, increasing their biological activity. VEGF induces the proliferation, differentiation, and migration of vascular endothelial cells [38,39]; increases capillary permeability [40]; and increases endothelial cell survival by preventing apoptosis [41,42]. In turn, VEGF secreted by endothelial cells can enhance the migration of tumor cells [43], protect them from apoptosis, and prevent anoikis through the activation of PI3K/AKT in HNSCC cancer stem cells [44]. In addition to all of these functions, VEGF has been shown in a number of preclinical studies to contribute to immune suppression. This immunosuppression can occur in several ways. Firstly, VEGF, by binding to VEGFR1 on stem cells of myeloid origin, prevents their differentiation into mature immune cells. Secondly, it induces the expression of the programmed ligand PD-L1 on antigen-presenting cells, which leads to a decrease in T cell activation [45,46].

*VEGF* expression is influenced by various factors in the TME, including hypoxia, growth factors, cytokines, and transcription factors. For example, enhanced tumor vascularization is provided by the dynamic interaction between endothelial cells, immune cells, and CAFs, which actively secrete pro-angiogenic factors [47,48,49]. Thus, TME macrophages, especially under hypoxic conditions, secrete TGF-β/-α, VEGF, IL-1, IL-6, and IL-8; these factors act as inducers of angiogenesis in HNSCC. For example, IL-8, IL-6, and EGF induce the phosphorylation of STAT3 and ERK in endothelial cells, which increases their survival and proliferation [50]. TGF-β, which is produced by many TME cells (CAFs, T regulatory lymphocytes, etc.) and is found in high percentages in HNSCC, also increases angiogenesis [51]. The high expression of angiogenic factors correlates with more advanced disease, resistance to conventional cytotoxic agents, and poor prognosis [22,52,53]. At least 90% of HNSCCs have increased expression of angiogenic factors such as VEGF. The overexpression of *VEGF* is associated with aggressive disease and poor outcomes in HNSCC [54,55]. In addition, the increased production of VEGF by tumor cells is associated with lymph node metastasis [56]. Aggarwal et al. [57] reported that serum VEGF levels were significantly higher in patients with oral squamous cell cancer and that these expressions were directly correlated with clinical stage evolvement and neck lymph node involvement. The interaction between HNSCC cells and endothelial cells triggers MAPK and Notch signaling, thereby promoting and enhancing tumor angiogenesis [58]. In a meta-analysis of 12 studies including 1002 patients with cancer of the oral cavity (70.8% of patients), pharynx (15.2%), and larynx (14%), *VEGF* expression was assessed, and its positivity was associated with a twofold increase in mortality after 2 years [22]. In addition, the overexpression of *TGF-β1* found in HNSCC ultimately leads to tumor growth and metastasis by facilitating angiogenesis [59]. Taking into consideration the important regulatory function of hypoxia in the initiation and enhancement of angiogenesis, in the next part, we will talk more about it and its role in HNSCC carcinogenesis.

## 3. The Role of Hypoxia in the TME of HNSCC

The TME is characterized by an inflammatory microenvironment that is also hypoxic, promoting increased angiogenesis [60]. Hypoxia is one of the key factors regulating the development of tumors of various types [61,62], including HNSCC [23]. Intratumoral hypoxia occurs in tumors that exhibit expansive properties, which reduces blood flow [63]. The oxygen concentration in tissues in HNSCC is about 1.3–1.9%, while in normal tissues, it is 5.3–6.7% [64]. The hypoxic microenvironment in the HNSCC contributes to the development of an aggressive carcinoma phenotype with a high metastatic rate, resistance to therapeutic agents, and higher tumor recurrence rates, leading to low therapeutic efficiency and poor outcomes [65,66]. Its presence in HNSCC is associated with resistance to radiation therapy since a hypoxic environment reduces the production of reactive oxygen species, which reduces radiation-induced DNA damage and makes cancer cells resistant to radiation [67]. Hypoxia is associated with chemoresistance in HNSCC and also promotes immune evasion by impairing the function of TAMs and T-lymphocytes [68]. In addition, hypoxia, characteristic of HNSCC, promotes epithelial–mesenchymal transition (EMT) and rapid metastasis [69].

The cellular response to hypoxia is realized through the signaling pathway of the transcription factor HIF (hypoxia-inducible factor) [70,71]. HIF is a heterodimer consisting of one oxygen-regulated isoform α subunit (HIF-1α, HIF-2α, or HIF-3α) and a constitutively expressed subunit HIF-1β (ARNT, the aryl hydrocarbon nuclear receptor translocator—a nuclear carrier of aromatic hydrocarbons) [72]. At sufficient oxygen concentrations, the de novo synthesized cytoplasmic subunit of HIF-α is regulated by the hydroxylation of proline residues with the help of prolyl hydroxylases (prolyl hydroxylase domain proteins—PHD1, PHD2, and PHD3), which promotes the proteasomal degradation of HIF using the E3-ubiquitin ligase von Hippel–Lindau complex (VHL) [73,74]. The activity of hydroxylases depends on the oxygen content in tissues: at low concentrations or in the absence of oxygen, it is suppressed, which leads to a decrease in the hydroxylation of HIF-α. As a result, the HIF-α subunit accumulates in the cytoplasm of cells and then translocates into the nucleus, where it dimerizes with the HIF-β subunit. In the nucleus, the HIF-α/β dimer interacts with hypoxia-responsive elements (HREs), which are located in the promoters of oxygen-dependent genes encoding proteins involved in systemic and cellular adaptation to hypoxia, such as erythropoietin, VEGF, etc. [75,76]. HIF regulates many cellular and molecular biological mechanisms of tumor development, including angiogenesis [77,78]. Tumor cells undergo adaptive and genetic changes that allow them to survive and proliferate in a hypoxic environment. During the development and progression of tumors, because of excessive cell proliferation and insufficient blood supply, hypoxia occurs, which activates angiogenesis, regulated by the HIF-dependent factor VEGF [62,77,79]. Oxygen deficiency induces an imbalance between the production of proangiogenic and antiangiogenic factors, which leads to the excessive, rapid, and chaotic formation of blood vessels with a significant increase in their number. Such vessels are characterized by thin walls and a variety of shapes and sizes [80]. HIF-1 controls the expression of several genes encoding angiogenic growth factors, including *VEGF*, *SDF1* (stromal-derived factor 1), *PGF*, *PDGFB*, and angiopoietin (*ANGPT 1* and *ANGPT 2*).

The expression of *HIF-1* and *HIF-2* in endothelial cells has different effects on vascularization. In experimental models, the inhibition of HIF-1 activity significantly suppresses the vascularization of tumors, particularly prostate cancer [78,81]. While the deletion of the *HIF1A* gene reduces tumor vascularization and growth, the deletion of HIF2A, conversely, enhances angiogenesis with the formation of immature vessels and an increase in the degree of tumor hypoxia [82,83]. Koukourakis et al. reported that *HIF-1/2α* overexpression was significantly associated with high microvessel density and *VEGF* expression in HNSCC [84]. Despite many clinical studies revealing that HIF-1α expression is linked to a poor prognosis in HNSCC, surprisingly, some studies have revealed the opposite. In surgically treated HNSCC patients, HIF-1α has been associated with improved disease-free and overall survival. The role of hypoxia and HIF in HNSCC was discussed in detail in a recent review [85].

Tumor progression is associated with metastasis, which is largely determined by the interaction of tumor cells with the microenvironment and includes the process of EMT [86]. HIF-1 regulates the process of tumor metastasis by changing the adhesion and motility of tumor cells and activating EMT processes. Hypoxia-induced EMT is characterized by a decrease in the expression of genes encoding proteins characteristic of the epithelium—E-cadherin and β-catenin—and an increase in the expression of genes encoding proteins of the mesenchymal phenotype—N-cadherin, vimentin, α-SMA (α-Smooth Muscle Actin), and chemokine receptor CXCR4 [87,88]. During metastasis, tumor cells lose their cell–cell adhesion capacity and acquire cell motility for invasion via EMT. In a majority of tumors, including HNSCC, EMT is tightly linked to hypoxia, leading to increased radioresistance [89,90]. Hypoxia that develops during tumor growth affects both tumor cells and the microenvironment. Hypoxia and HIF activation regulate tumor vascularization, changes in metabolism, cell survival, and death. Hypoxia promotes EMT, leading to tumor cell migration and cancer-stem-cell-like properties, including resistance to treatment.

## 4. CAFs in HNSCC

**Figure 2:**
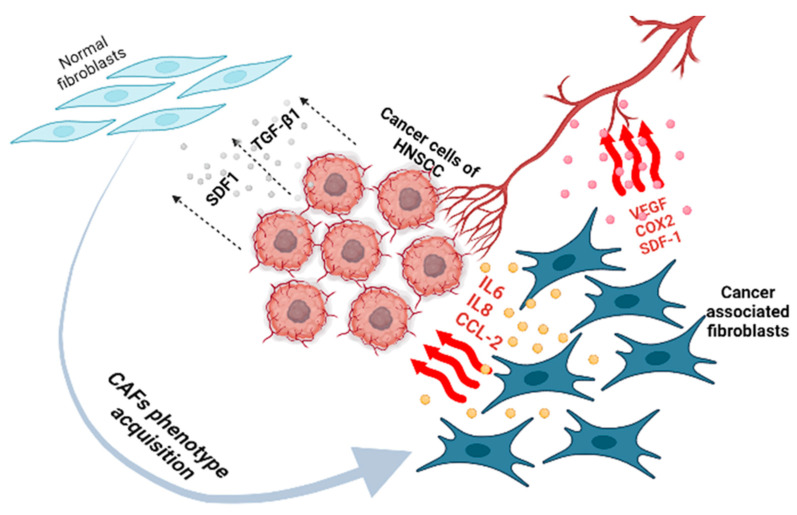
CAFs in HNSCC: phenotype acquisition and contribution to cancer cell maintenance and angiogenesis.

Fibroblasts are the most numerous connective tissue cells of mesenchymal origin [91] involved in the synthesis and degradation of ECM components, are responsible for ensuring the structural integrity of most tissues, produce basement membrane components that provide a protective barrier around epithelial tissue, and determine the polarity and functioning of epithelial cells [92,93]. CAFs are one of the main components of the stroma, and they can account for up to 80% of all tumor cells [94]. In tumors, they are heterogeneous and can be differentiated from various cell types (mesenchymal stem cells, fibrocytes, adipocytes, pericytes, and epithelial cells) through EMT [94,95,96]. However, the main sources of CAFs in the HNSCC TME are local normal fibroblasts (NFs). Tumor cells influence the differentiation of NFs into CAFs, and CAFs influence metastasis and tumor progression. So, tumor cells constantly produce different growth factors such as transforming growth factor-beta1 (TGF-β1) and stromal cell-derived factor-1 (SDF1), interleukin (IL) 1β, platelet-derived growth factor A (PDGF-A), and fibroblast-activating protein and platelet-derived growth factor, which can facilitate the transformation of progenitor cells or NFs into CAFs [97,98,99] (Figure 2) [100,101]. In turn, CAFs secrete cytokines and ECM components, thereby remodeling the TME and providing a favorable environment for the growing tumor [102]. CAFs play a major role in carcinogenesis and can regulate proliferation, invasion, and metastasis by changing the production of cytokines, chemokines, and the ECM in the TME of HNSCC [99,103,104,105].

In HNSCC, CAFs differ from NFs by expressing more than 500 genes encoding proteins such as IGF-2, IL-6, IL-8, and CXCL-1, etc. [106,107]. With regard to functional characteristics, CAFs produce growth factors such as epidermal growth factor (EGFR) and VEGF and also produce matrix metalloproteinases (MMPs), which are involved in the remodeling of the ECM and promote tumor growth and metastasis [108,109].

### 4.1. Contribution of CAFs to HNSCC Progression

CAFs can activate tumor cell proliferation and growth, tumor invasion, and metastasis (Table 1) and influence other TME cells by producing a variety of growth factors and cytokines, such as hepatocyte growth factor (HGF), fibroblast growth factors FGF2 and 7, TGF-β, PDGF, osteopontin (OPN), SFRP2 (secreted frizzled-related protein 2), SDF-1 (stromal cell-derived factor 1), VEGFA, IL-6, IL-8, and many others [96,109,110,111]. Thus, CAFs are involved in tumor angiogenesis and regulate blood flow [112]; the SDF-1 released by CAF stimulates tumor neovascularization through the recruitment of endothelial progenitor cells [113] and also activates the proliferation of tumor cells [113]. In addition, CAFs secrete ROS, which damage the DNA of surrounding cells, which determines their promutagenic properties [114].

### 4.2. CAFs and Cancer Cell Proliferation

CAFs activate the proliferative activity of tumor cells in HNSCC. According to [115], in OSCC, CAF-derived microfibrillar-associated protein 5 (MFAP5) activates the phosphorylation of PDK1 and Akt and inhibits cRAF and PTEN, which leads to an increase in the proliferative capacity of tumor cells. It was shown that, in HNSCC, these cells produce IL-6, which regulates OPN expression through STAT3 activation, which may promote tumor proliferation [116]. The production of chemokines such as CCL-2 by CAFs leads to the activation of proliferation, as well as the migration and invasion of tumor cells in oral squamous cell cancer (OSCC) [117]. CAFs can also support tumor cell stemness through the synthesis of insulin-like growth factor (IGF)-II [118]. CAFs secrete carboxypeptidase E, PDGFD, EFEMP1 (EGF-containing fibulin ECM protein 1), and IBP5 and IBP7 (insulin-like growth factor binding proteins 5 and 7), which exert their effects on tumor cells through IGF and PDGFR and activate cancer stem cell proliferation [119].

### 4.3. CAFs and Metastasis

CAFs promote tumor cell metastasis through the formation of transient heterotypic intercellular contacts and the production of paracrine action factors [120]. CAFs can facilitate invasion through the generation of tracks in the ECM by digesting the matrix, thereby allowing cancer cells to leave the site of origin [109,121,122,123]. This process involves podoplanin (PDPN), Rho-ROCK (Rho-associated protein kinase), and MMPs, which are expressed by CAFs [124,125]. The activation of EMT is one of the main processes leading to tumor invasion and metastasis. In cells subjected to EMT, there is usually an activation of mesenchymal markers such as vimentin, fibronectin, and N-cadherin and a concomitant decrease in the activity of cell–cell contact maintenance proteins such as E-cadherin, occludins, and claudins, which impair cell adhesion to each other and promote migration and invasion [123]. 

Recently, many studies have shown that CAF-secreted factors like IL-6, OPN, HGF, and CXCL12 in different cancers are involved in EMT [123]. CAFs produce high levels of TGF-β1, which can upregulate vimentin, SNAIL, ZEB2, or long noncoding RNAs (lncRNAs) and downregulate E-cadherin [126,127]. One of the possible mechanisms of CAFs’ EMT activation in tumor cells and, as a consequence, metastasis, is a STAT3-mediated reduction in E-cadherin expression. It is known that one of the EMT transcription factors is ZEB1, linked in a feedback loop with mir-200 [128]. STAT3, which is overexpressed in CAFs, can bind directly to the ZEB1 promoter and induce its expression; in turn, ZEB1 represses E-cadherin, which is a feature of EMT promotion and invasion [129]. In squamous cell carcinomas, the interaction of N-cadherin, located on the surface of CAFs, with tumor cell E-cadherin promotes tumor invasion through the activation of the β-catenin–vinculin signaling pathway and, as a consequence, actin remodeling in both types of migrating cells [130].

Co-culturing CAFs, rather than normal fibroblasts, with OSCC tumor cells has been shown to activate their proliferation and migration [131]. In another study, the authors found a relationship between the overexpression of periostin by CAFs and the proliferative activity and migration of HNSCC tumor cells [132]. Periostin is a secreted ECM protein encoded by the POSTN gene, which, among other things, enhances tumor metastatic activity and stimulates the formation of tumor stem cells in HNSCC [133].

Another factor that is a product of CAFs and can significantly influence the formation of HNSCC metastases is BDNF (brain-derived neurotrophic factor). The activation of the BDNF-TrkB (neurotrophin receptor tyrosine kinase B) signaling pathway can lead to the progression of HNSCC [134]. TrkB induces EMT in tumor cells and plays an important role in invasion in HNSCC [135].

CAFs have been shown to produce prostaglandin E2 (PGE2), which not only stimulates the proliferation of HNSCC tumor cells, inhibits apoptosis, and determines the development of immunosuppression in the TME but also activates the invasive properties of tumor cells through interaction with receptors (EP1, EP2 and EP3) expressed by tumor cells [136,137].

Greater CAF density in the TME is associated with higher rates of perineural invasion (PNI) in HNSCC. Perineural invasion, in which tumor cells infiltrate the nerve sheath, mediates a distinct form of metastatic spread in HNSCC [138]. By producing various proangiogenic factors, CAFs can influence neovascularization in the tumor, which is very important for tumor progression. Thus, it has been shown that CAFs express PDGFR (platelet-derived growth factor receptor), which activates not only tumor cell proliferation but also angiogenesis [139]. The activation of angiogenesis in HNSCC is associated with the COX-2-mediated production of prostaglandin E2 (PGE2) by CAFs [107,140].

CAFs in NPC (nasopharyngeal carcinoma) are characterized by high levels of α-SMA expression, and the tumor stroma contains an increased number of endothelial progenitor cells, which promote vascular growth through the activation of VEGF- and SDF-1 [141]. In turn, the overexpression of TGF-β1 observed in HNSCC promotes angiogenesis and epithelial hyperproliferation [142].

HNSCC tumor cells produce IL-6, CXCL8, and epidermal growth factor (EGF), which improve the survival and angiogenic potential of endothelial cells through the activation of the STAT3/protein kinase B (AKT)/extracellular signal-regulated kinase (ERK) signaling pathways [50].

### 4.4. CAFs and Angiogenesis

By producing different proangiogenic factors, CAFs can induce neovascularization in the tumor, which is very important for energy metabolism and tumor progression. Thus, it has been shown that CAFs express PDGFR, which activates not only tumor cell proliferation but also angiogenesis [139]. The activation of angiogenesis in HNSCC is associated with the COX-2-mediated production of prostaglandin E2 (PGE2) by CAFs [140,143]. CAFs in NPC are characterized by high levels of α-SMA expression, and the tumor stroma contains an increased number of endothelial progenitor cells, which promote the activation of VEGF and SDF-1 [141]. In turn, the overexpression of *TGF-β1* observed in HNSCC promotes angiogenesis and endothelial proliferation [142] because it is known that TGF-β1 activates Smad3 phosphorylation and regulates *TNC, CDKN1A*, and *CDKN2A* expression in order to promote proliferation of endothelium progenitor cells [144]. HNSCC tumor cells produce IL-6, CXCL8, and EGF, which improve the survival and angiogenic potential of endothelial cells through the activation of the STAT3/AKT/ERK signaling pathways [50].

### 4.5. CAFs and Immune Response

CAFs release cytokines and chemokines that regulate the migration, differentiation, and functional polarization of immune cells in the TME. According to [145], this inhibits T cell proliferation in OSCC, leading to the development of an immunosuppressive state in the TME. Moreover, in HNSCC, the increased expression of AKT3 by CAFs also activates the development of immunosuppression, which correlates with poor prognosis [146].

In vitro, it was found that CAFs suppress the functions of T-lymphocytes more than NFs: CAFs reduce proliferation and induce the apoptosis of T cells and Treg lymphocytes [147]. In addition, cytokines produced by CAFs promote the recruitment of tumor-associated macrophages and shift their differentiation toward the anti-inflammatory M2 subtype, which is correlated with poor outcomes in HNSCC [145,148,149]. Thus, CAFs lead to the development of an immunosuppressive state in TME cells and avoid tumor immunosurveillance.

### 4.6. CAFs and Metabolic Activity

CAFs can promote tumor cell growth by regulating their glucose, amino acids, and lipid metabolism and mitigate the development, invasion, and resistance of tumor cells to therapy [150,151,152,153]. Cancer metabolic activity is high, and its cells actively consume glucose even under aerobic conditions, thereby increasing lactate secretion [99]. This effect is called the “Warburg effect” or “aerobic glycolysis” [154]. However, tumors can also use lactate and glutamate as energy [155]. During carcinogenesis, in relationships with tumor cells, CAFs change their metabolism, which leads to metabolic symbiosis, in which tumor cells use CAF metabolites as an energy source. When exposed to tumor cells, CAFs exhibit tumor-like aerobic glycolysis, known as the inverse “Warburg effect” [151]. As a result, CAFs can secrete products such as pyruvate and lactate, which are used by tumor cells to maintain their metabolism [99]. Zhang et al. [156] showed that the stromal CAFs of OSCC express high levels of integrin subunit beta 2 (ITGB2), which correlates with poor prognosis. ITGB2 is known to regulate the PI3K/AKT/mTOR signaling pathway, which increases the glycolytic activity of CAFs and the generation of lactate. This leads to proliferation of OSCC tumor cells.

Researchers have discovered the lactate shuttle phenomenon in nasopharyngeal carcinoma (NPC) [150]. The inhibition of monocarboxylate transporter 4 (MCT4) in activated CAFs has been shown to result in the decreased proliferation, invasion, and colony formation of NPC cells. Moreover, Kumar D. et al. [157] found that CAF-secreted HGF promotes HNSCC progression. It has been shown that CAFs secrete HGF, which leads to tumor cells switching to glycolysis, and tumor cells secrete FGF to “force” CAFs to consume lactate.

CAFs can alter the availability of metabolites in the TME. For example, they can affect the bioavailability of tryptophan and arginine, which are essential for the activity and functionality of T-lymphocytes. Thus, metabolic competition between CAFs and immune cells can lead to a decrease in the antitumor activity of T cells [110]. Thus, CAFs are potential targets for antitumor agents, so they can express different cytokines and the growth-factor-induced activation of the angiogenesis, proliferation, invasion, and metastasis of HNSCC. To overcome drug resistance or stop tumor progression, it could be possible to target genes like *TGF-beta*, *MMP9*, *HGF*, *OPN*, *CXCL12*, etc.

## 5. Role of the ECM in HNSCC

As discussed above, HNSCC is composed of a collection of malignant epithelial cells and TME cells (e.g., fibroblasts, vascular cells, immune cells) that secrete ECM proteins and numerous signaling molecules. Although the initiating genomic changes associated with HNSCC occur in epithelial cells, the changes that occur in the TME facilitate tumor progression and provide various adaptation mechanisms when exposed to damaging factors from the outside (anticancer drugs, radiation therapy) [158]. The ECM is involved in virtually all stages of carcinogenesis, including cancer cell proliferation and invasion, angio- and lymphangiogenesis, the evasion of immune responses, the creation of an immunosuppressive environment, metastasis, and resistance to anti-tumor therapies [159]. The ECM is one of the main components of intercellular and intertissue interactions, providing signaling activity. It has a supporting and shaping function, providing the characteristic shape and size of organs and tissues [160,161]. The ECM is composed of fibrillar and non-fibrillar collagens, elastic fibers, and glycosaminoglycans (GAGs). The ECM is a key structural component of any normal and pathological tissue [12]; in turn, persistent disruption of its homeostasis can lead to the development of various pathological conditions, including cancer. In tumor tissue ECM undergoes changes—its composition, organization, and mechanical properties are altered. The altered composition of the ECM of tumors affects cancer cells, ensures cell insensitivity to growth inhibitors, promotes angiogenesis, and protects against antitumor agents [158]. Many solid tumors are characterized by an abundance of various ECM molecules, such as fibrillar collagens, fibronectin, elastin, laminins, and hyaluronic acid, with the predominance of one or another component depending on the type of cancer [162,163,164]. Recently, the ECM has been assigned one of the main roles in the oncogenesis, metastasis, and progression of malignant neoplasms [165]. An altered ECM composition is characteristic of most solid tumors. At the same time, the predominance of one or another ECM component depends on the anatomical location of the tumor, the causative factors of its origin, and the stage of the disease. [166]. In addition, tumor ECM is biochemically distinct from healthy tissue. Tumor stroma is usually stiffer than normal tissue stroma (~400 Pa versus 150 Pa, respectively) [167,168]. At the same time, the increase in stiffness and the remodeling of the ECM begin to be traced at the stage of precancerous changes, which, in turn, also contribute to the malignant transformation of cells. The dynamic interaction during carcinogenesis between tumor cells, microenvironmental cells, and the ECM leads to aberrant mechanotransduction and further malignant transformation [169]. 

The ECM of HNSCC is a collection of molecules with a variety of cytokines, intermediate metabolites, nutrients, hormones, and chemokines secreted by tumor and TME cells [170]. The ECM promotes the adhesion and migration of cancer cells, which causes tumor progression and metastasis (Table 2). Given its ability to bind secreted factors, the ECM is considered a functional bioregulatory platform on which the processes of carcinogenesis can take place [171]. Because HNSCC represents a diverse and complex set of diseases, it exhibits a high level of ECM heterogeneity. Indeed, malignancies arising in histologically distinct mucosal squamous epithelia such as the oral cavity, larynx, hypopharynx, and oropharynx may exhibit distinct stromal features. In oral squamous cell carcinoma, single-cell mRNA profiling has revealed a prominent stromal compartment with large numbers of matrix-producing CAFs in the majority of tumors examined [172]. The active production of ECM components by CAFs leads to increased tumor stiffness, which activates oncogenic intracellular signaling pathways, such as the β-catenin, Akt, PI3K, and focal adhesion kinase (FAK) pathways, and suppresses tumor suppressor genes such as phosphatase and tensin homolog [173,174].

In HNSCC, signaling between malignant epithelial cells and stromal cells causes increased activity in ECM components that control carcinoma cell migration, modulate the cytokine milieu, and promote immune evasion in tumors [170]. During HNSCC carcinogenesis, changes also occur in the ECM: its composition and density are altered. In the early stages of HNSCC progression, nascent transformed epithelial cells induce a wound-healing-like response marked by the activation of stromal fibroblasts and the strong accumulation of ECM and ECM-related proteins collectively known as matrisomes [175]. CAFs are the main matrix-producing cells of the stroma. In a proteomic analysis of the ECM produced by CAFs isolated from HNSCC, collagens I, III, VI, and XII; fibronectin; tenascin C; and TGF-β-induced (TGFBI) fibrillin were among the major core components [176]. Proteins associated with the ECM deposited by these CAFs include galectin-1, annexin A2, the ECM regulator SERPINH1, tissue transglutaminase (TG2), HTRA1, and lysyl oxidase homolog 2 (LOXL2), while secreted factors associated with the ECM included insulin-like growth factor 2, several alarmins, Wnt5A, and FGF [170].

As mentioned above, stromal cells (to a greater extent) and cancer cells participate in the production of ECM. Reciprocal epithelial–mesenchymal interactions at the tumor–stroma interface enhance the expression of cancer cell matrisomal proteins and their incorporation into the ECM. To demonstrate the important role of these components in tumor progression, a proteomic study of pancreatic ductal adenocarcinoma was conducted, which showed that high levels of tumor-cell-derived matrisomal proteins correlate with poor patient survival [177].

Let us consider changes in some ECM components of HNSCC. As with other types of cancer, with HNSCC, there is a thickening of the ECM, in which collagen plays a significant role. During tumor growth, increased interstitial collagen deposition is accompanied by fiber reorganization and enzymatic cross-linking (lysyl oxidases and lysyl hydroxylases), which correlates with increased tumor invasive potential and poor patient survival [178,179]. To classify changes in collagen arrangement that accompany carcinoma progression, distinct patterns of fibrillar collagen organization, called “tumor-associated collagen signatures,” have been defined [180]. Also, collagen in the HNSCC ECM activates the tyrosine kinase DDR1 receptor in tumor epithelial cells, which triggers pro-tumor activity. Moreover, increased DDR1 expression is associated with worse overall survival [181]. DDRs (discoidin domain receptors) can spontaneously bind to collagen and are not regulated by intracellular or extracellular signals [182,183]. The issue of DDR expression in various cancers remains controversial today, but there is evidence of increased DDR expression in cancer. For example, the overexpression of DDR1 has been observed in HNSCC [184]. Lysyl oxidase-like 2 (LOXL2) is a member of the lysyl oxidase (LOX) family of secretory enzymes, which are lysine deaminases that cross-link ECM proteins such as collagen [185,186]. Increased levels of LOXL2 have been found in HNSCC tissue [187]. The increased expression of LOXL2 mRNA has also been detected in metastatic lesions of HNSCC [188].

Let us review the major glycoproteins of the ECM of HNSCC. Fibronectin (the major glycoprotein of the ECM) is significantly overexpressed in patients with this type of cancer and has been obviously correlated with higher pathological stages and poor prognosis [180,189]. The downregulation of this glycoprotein suppresses the proliferation, migration, and invasion of HNSCC cells and inhibits macrophage M2 polarization in vitro [189]. Another HNSCC ECM glycoprotein, tenascin C, which is involved in the modulation of the immune response in many diseases [190], is also subject to changes. Even in the last century, a number of studies have described the role of tenascin in carcinogenesis: it induces cell migration [191], angiogenesis [192], and the expression of MMPs [193], which themselves are involved in promoting tumor growth and invasion [194]. Tenascin C has also been shown to promote tumor progression in a carcinogen-induced immunocompetent mouse model of OSCC by stimulating the formation of an immunosuppressive stroma [195]. Laminin expression is also upregulated in HNSCC. Laminin-5 hyperexpression is associated with high rates of HNSCC budding, suggesting that it is associated with the establishment of an invasive phenotype [196].

Integrins are transmembrane heterodimers consisting of α- and β-subunits. Integrins can bind collagen [197] and bind to various proteins, such as fibronectin, fibrinogen, laminin, and vitronectin [198]. Integrins also connect the ECM to the intracellular actin cytoskeleton. In addition, integrins provide the process of mechanotransduction: integrins perceive the mechanical force of the ECM and then transmit signals to intracellular proteins, such as tyrosine kinases FAK and Src. The activation of αvβ3 integrin correlates with poor prognosis for patients with OSCC [199,200]. Integrin α5 (ITGA5) promotes the proliferation, migration, and invasion of HNSCC cells by regulating the activation of the PI3K/AKT signaling pathway, and increased expression is associated with poor prognosis [201]. The overexpression of integrin αvβ6 is observed in HNSCC and correlates with invasive potential and progression [199,202]. 

Perlecan is a heparin sulfate proteoglycan that has five domains and is one of the main components of the ECM, participating in cell proliferation and differentiation (through interaction with integrins) [203]. It plays an important role in lipid metabolism, inflammation, wound healing, thrombosis, and cancer angiogenesis [204]. In HNSCC, perlecan promotes tumor cell growth, chemoresistance, migration, and invasion, mainly by regulating heparin-binding growth factors such as FGF-2, VEGF-A, and Hedgehog (Hh) [159,205]. Periostin is another important proteoglycan of tumor ECM. According to one study [132], the overexpression of periostin can also be observed in HNSCC, which is associated with tumor proliferation and metastasis. 

The ability of tumor cells and TME cells to synthesize ECM components critically influences tumor progression [206]. Understanding the nature of heterotypic interactions between tumor cells, the ECM, and CAFs in the TME will provide insight into the mechanisms underlying tumor progression and metastasis and identify new targets for antitumor agents. The densified structure of the ECM, observed in many types of cancer, determines tumor progression both by creating barriers to the entry of therapeutic agents and by creating certain conditions in the tumor tissue itself. Today, it is known that the densified structure of the ECM observed in various types of cancer leads to a loss of sensitivity to anticancer drugs [207] and radiation therapy [208]. In addition to creating a “protective shield” effect that is difficult for antitumor agents to overcome [209,210], dense ECM compresses blood vessels, which also prevents drugs from reaching tumor cells. The compression of blood vessels by dense ECM leads to local hypoxia [211,212], which, in turn, activates antiapoptotic pathways and stimulates neoangiogenesis [213]. Immune cells migrating toward tumor cells along a cytokine concentration gradient cannot reach the tumor because they will encounter a dense ECM. Thus, ECM density regulates the process of the infiltration of immune cells into tumor tissue [210,214]. Hypoxia and metabolic stress, which are a consequence of high ECM density, lead to an increase in the content of immunosuppressive factors IL-10, CCL18, CCL22, TGF-b, prostaglandin-E2, and VEGF-A [213,215,216]. In this case, TGF-b attracts T-reg cells into the tumor [217] and acts as an M-2 polarizer for macrophages [218]. Dense ECM induces the transition of tumor tissue cells into cancer stem cells (CSCs), which, in turn, actively proliferate in hypoxic environments. In addition, a number of studies have demonstrated the resistance of CSCs to anticancer drugs [169,219,220]. During HNSCC carcinogenesis, ECM destabilization occurs. The increased deposition of a number of proteoglycans and collagens leads to ECM remodeling. Remodeled ECM causes disruption in cell polarity and enhances growth factor transport, causing biochemical and biomechanical changes. These changes ultimately contribute to the metastatic cascade: cell migration into the interstitial matrix and then into the vasculature is stimulated [159,221].

The ECM (together with the basement membrane) is a barrier that tumor cells must overcome on the “pathway to vascular invasion” [222,223]. In this case, basal membrane disruption is defined as a critical event of tumor invasion that marks the beginning of the metastatic cascade [224,225]. The surface of HNSCC cancer cells is characterized by the presence of invadopodia, which play an important role in the process of tumor invasion. Invadopodia mediate tumor dissemination by degrading ECM-restrictive proteins with matrix MMPs [102]. MMPs are members of a family of calcium- and zinc-dependent endopeptidases that degrade other components of the ECM and thus ensure its constant renewal [226]. In total, about 24 members of the MMP family have been identified in humans. In addition to the above-mentioned function, MMPs destroy the basal membrane and capillary wall and stimulate neoangiogenesis; therefore, these enzymes have a key role in the progression of HNSCC [227,228,229]. A number of studies have demonstrated an increase in MMPs in HNSCC [230,231]. Interestingly, the level of MMP elevation depends on the anatomical localization of squamous cell cancer in the head and neck region.. For example, MMP1 and MMP10 are highly expressed in OSCC [232] and MMP3 expression is elevated in squamous cell carcinoma of the tongue [233]. The activity of MMP14, MMP2, and MMP9 in the ECM is significantly increased in HNSCC cell lines with high metastatic potential, as well as in samples from patients with oral cancer with lymph node involvement [234,235]. MMP14 plays a key role in the early stages of tumor invasion and cancer progression. MMP14 is concentrated on the surface of cancer cell invadopodia and destroys a number of VSMC components: collagen types I, II, and III; fibronectin; tenascin; and perlecan. A recent study [236] demonstrated an association between MMP14 levels and the extranodal extension of OSCC. In addition, ECM metalloproteinase inducer (EMMPRIN, also known as CD147) is an additional factor involved in tumor invasion and metastasis. In hypopharyngeal squamous cell carcinoma, CD147 appears to mediate ECM degradation by stimulating the synthesis of MMPs and promote angiogenesis by stimulating *VEGF* expression [237]. One study [238] showed that MMP3 can be used as a potential biomarker for oral cancer progression. In addition, a recent study [231] found that MMP family expression correlates with the levels of infiltration of six immune cells, B cells, CD8 + T cells, CD4 + T cells, macrophages, neutrophils, and dendritic cells, suggesting that the MMP family may reflect immune status and serve as a prognostic sign in HNSCC. Kudo et al. demonstrated that the highly invasive HNSCC cell line MSCC-inv1 significantly overexpresses MMP19 [239]. MMPs have the molecular function of modulating a number of latent signaling proteins located in the ECM, including cytokines and growth factors such as resting TGF-β, which forms a complex with TGF-β-binding protein-1 in the ECM. Thus, TGF-β modulates MMP expression, resulting in a bidirectional regulatory loop that enhances TGF-β signaling and promotes cancer progression [240]. The MMP family can be used as therapeutic targets and prognostic biomarkers for HNSCC depending on their role in the disease. Some scientists have used sesamin extracted from the sesame oil of pepper bark to regulate MMP2, thereby inhibiting HNSCC migration and invasion [241]. Another team of researchers showed that mulberry leaf extract can inhibit the activity of MMP2 and MMP9 and inhibit the migration and invasion of HNSCC [242]. Considering the multicomponent nature of the ECM, not all possible roles of its components in the development and progression of HNSCC have been described to date. Increased knowledge about the role of ECM components in HNSCC will help guide the search for new diagnostic and treatment options.

**Table 2:** Role of ECM components in the progression of HNSCC.

Main Components of the HNSCC ECM		What Are the Effects on HNSCC Progression?
Collagens		- Create migration pathways for cancer cells and affect their invadopodia [243], thereby promoting invasion and metastasis [244]. - Promote PI3 kinase (PI3K) activity and induce invasion [245]. - Induce resistance to epidermal growth factor receptor tyrosine kinase inhibitors (EGFR-TKIs) through the activation of mTOR via the AKT-independent pathway [246]. - Promote the proliferation and invasion of cancer cells via the activation of the mitogen-activated protein kinase/extracellular signal-regulated kinase (MEK/ERK) signaling pathway [247].
Elastin		- A positive correlation has been observed between elastin degradation and the degree and stage of the disease [248].
Fibronectin		- Takes part in the transformation of cancer cells into stem cancer cells; stimulates their growth, proliferation, and evasion of growth suppressors. - Stimulates angiogenesis by increasing the content and transmission of VEGF-mediated signaling [249]. - Provides resistance of cancer cells to anoikis through a mechanism involving fibronectin and αv/FAK integrin receptor signaling [250].
Laminins		- Activate EGFR/MAPK signaling [251]. - High expression is positively correlated with tumor = invasive potential and poor prognosis [252,253,254].
Hyaluronan		- Participates in the activation of the migration, survival, and chemoresistance of cancer stem cells [255].
Tenascin-C		- Stimulates proliferation, migration, angiogenesis, and metastasis [256]. - Induces epithelial–mesenchymal transition. - Induces and activates other signaling pathways in cancer cells such as JNK, Wnt, Notch, AKT/HIF1α, and TGF-β. - Creates barriers for T-lymphocytes to enter the tumor.
Integrins		- Provide radioresistance [257]. - Integrin αvβ3 is actively involved in tumor angiogenesis [258]. - Overexpression of αv integrin subunit is associated with tumor invasion and metastasis [259]. - Participate in HNSCC proliferation and invasion by activating the mitogen-activated protein kinase/extracellular signal-regulated kinase (MEK/ERK) signaling pathway [247]. - In nasopharyngeal carcinoma, the mRNA and protein expression levels of integrin αv also correlate with tumor size and lymph node spread [260].
MMPs	MMP2	- Increased expression of MMP 2 in HNSCC is positively correlated with aggressive invasion, poor survival, and metastasis to lymph nodes.
	MMP9	- Destroys type IV collagen, a major component of the basal membrane, promoting invasion and metastasis [260]. - Enhances the bioavailability of VEGF, thereby increasing the amount of VEGF in the tumor microenvironment [261]. - MMP9 overexpression is associated with tumor recurrence, metastasis to lymph nodes, and the development of secondary primary cancer [262].
	MMP13	- Increases the secretion of VEGF-A [263].
	MMP14	- Overexpression is associated with extranodal spread and is associated with poor prognosis [236].
	MMP17	- Assists in cell invasion. - Stimulates movement of amoeboid cells in tumor tissue and invadopodia [264]. - Induced by HIF1-⍺-mediated hypoxia and enhances metastasis [264].
	MMP20	- Promotes EMT; facilitates migration of cancer cells across the basal membrane [265].
Perlecan		- Involved in adhesion and migration of cancer cells. - Overexpression is associated with resistance of HNSCC cancer cells to cisplatin [266].

## 6. Conclusions

The progression of HNSCC is a complex process involving a series of consecutive and overlapping events. Among these events, the main ones are the proliferation and invasion of the tumor into adjacent tissues, the formation of new vessels, intravasation, cancer cell survival, escape from anti-tumor immune responses, extravasation, and the colonization of other organs. The TME is a key component of this cascade of events, as it is involved in phenotype changes (cancer cells acquire a stem cell phenotype, immune cells acquire a pro-tumor phenotype) and the modulation of the behavior of both tumor cells and all cellular components of tumor tissue. Analyzing the relationships between TME components, one gains an impression of some “vicious circles” that condition the aggressive potential of HNSCC. Understanding the peculiarities of heterotypic interactions between cancer cells, new vessels, CAF, ECM, and hypoxia will allow us to understand the mechanisms mediating rapid tumor progression and its resistance to antitumor drugs and radiation therapy. Basic research aimed at studying the role of TME components in HNSCC carcinogenesis may serve as a key to the discovery of both new biomarkers–predictors of prognosis and targets for new antitumor strategies.

## Figures and Tables

**Table 1:** CAF involvement in different stages of HNSCC progression.

Process	CAFs Function
Proliferation	- CAFs promote tumor proliferation via the activation of PDK1 and Akt phosphorylation and the inhibition of cRAF and PTEN. - CAFs induce tumor proliferation through the activation of STAT3 and CCL-2. - CAFs promote tumor proliferation through IGF and PDGFR.
Metastasis	- CAFs produce PDPN, Rho-ROCK, and MMPs that remodel the EMC and promote tumor cell metastasis. - CAFs produce high levels of TGF-β1 that can upregulate EMT transcription factors—vimentin, SNAIL, ZEB2, or lncRNAs—and downregulate E-cadherin, resulting in mitigated invasion.
Immune response	- CAFs inhibit T cell proliferation, activate Treg, and lead to immunosuppression.
Angiogenesis	- CAFs activate neoangiogenesis by producing VEGF - COX-2-mediated production of prostaglandin E2 (PGE2) via CAFs induces angiogenesis.

## Data Availability

Not applicable.
